# Petroleomics *via* Orbitrap mass spectrometry with resolving power above 1 000 000 at *m*/*z* 200†

**DOI:** 10.1039/c7ra12509g

**Published:** 2018-02-07

**Authors:** Eduardo M. Schmidt, Marcos A. Pudenzi, Jandyson M. Santos, Celio F. F. Angolini, Rosana C. L. Pereira, Ygor S. Rocha, Eduard Denisov, Eugen Damoc, Alexander Makarov, Marcos N. Eberlin

**Affiliations:** ThoMSon Mass Spectrometry Laboratory, Institute of Chemistry, University of Campinas, UNICAMP Campinas 13083-725 SP Brazil eberlin@iqm.unicamp.br eduardomschmidt@yahoo.com; Nova Analítica Importações e Exportações LTDA 04131-000 São Paulo, SP Brazil; Petróleo Brasileiro S/A, PETROBRAS, CENPES 21941-915 Rio de Janeiro, RJ Brazil; Thermo Fisher Scientific Hanna-Kunath-Strasse 11 28199 Bremen Germany alexander.makarov@thermofisher.com

## Abstract

The performance of the high-field MegaOrbitrap Fourier transform mass spectrometer (FT-MS) with electrospray ionization (ESI) was evaluated to perform petroleum sample characterization *via* classical petroleomics approaches. Pertinent parameters that underpin the main figures of merit, that is, signal to noise ratios, dynamic range, spectral error, scan speed, mass accuracy and mass resolving power = *R*_p_, and provide subsidies to develop these analyzers were tested. Comparisons are made with data obtained using the most common petroleomics instrument, which is a Fourier transform ion cyclotron resonance mass spectrometer (FT-ICR MS), that has been used in the last decade in our laboratory for crude oil analysis providing *R*_p_ of 340 000 at *m*/*z* 400 with transients of 3 s duration, and has been extensively demonstrated to fulfill all major requirements for precise petroleomics investigations. The high-field compact MegaOrbitrap mass analyzer, when operated at an *R*_p_ = 840 000 at *m*/*z* 400 (*R*_p_ > 1 000 000 at *m*/*z* 200) with a detection time of 3 s, was found to be well suited for adequate characterization of crude oil. Accurate class classification and mass accuracy below 1 ppm was obtained leading to proper, comprehensive petroleomics characterization.

## Abstract 2

The performance of the high-field MegaOrbitrap Fourier transform mass spectrometer (FT-MS) with electrospray ionization (ESI) was evaluated to perform petroleum sample characterization *via* classical petroleomics approaches.

## Graphical abstract

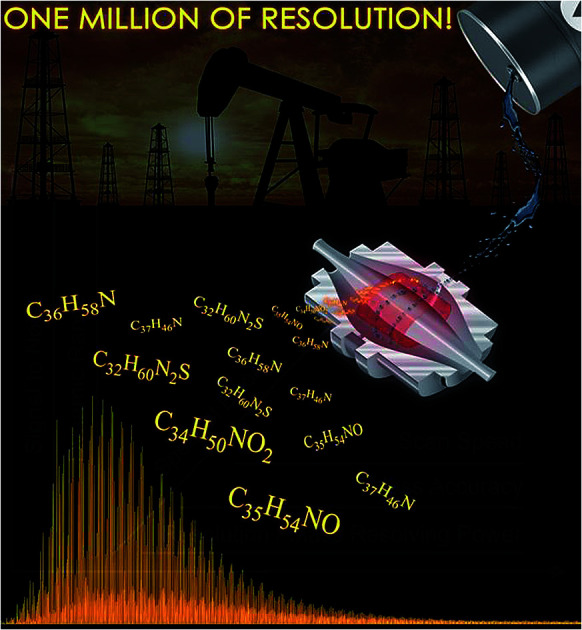

## Introduction

The measure of mass with high resolving power (*R*_p_) and mass accuracy to unambiguously determine molecular formulae and accurately define isotopic signatures have been central to mass spectrometry analysis. The continuous pursuit for higher *R*_p_ and accuracy has been recently driven by the increasing demands of the “omics” fields, especially in proteomics^1,2^ and petroleomics.^3,4^ Due to its great complexity in terms of molecular composition, the direct analysis of crude oil by MS without previous chromatographic separation must rely on the ability of the mass spectrometer to separate many thousands of ions, demanding high accuracy and ultrahigh *R*_p_ normally not lower than 400 000 at *m*/*z* 400 (400 000 at 400). Novel cell designs and higher field magnets for Fourier transform ion cyclotron resonance mass spectrometry (FT-ICR MS)^5,6^ are key examples of the MS race towards ultrahigh *R*_p_ and accuracy. FT-ICR has been so far the gold standard, actually the only choice for petroleomics investigations since it has been the only instrument able to provide the necessary ultrahigh *R*_p_ and accuracy to separate and attribute the myriad of isobaric ions faced in this field. For instance, a famous isobaric doublet is formed by molecules differing by C_3_*versus* SH_4_ in their formula which leads therefore to a mass difference as little as 0.00337 Da.^4^ But dependence on cryogens (liquid helium and in some cases liquid nitrogen), the need for magnets of higher field and cost, logistic constraints in their transportation and installation due to large size dimensions have been major FT-ICR MS drawbacks.^2^ Looking for alternatives, a “zig zag” multireflecting TOF analyzer has been tested and demonstrated to offer a reasonable platform for petroleomics MS but the limited *R*_p_ of the instrument (100 000 at 400) still led to some class misassignments.^17^

The Orbitrap orbital electrostatic trap analyzer based on Kingdon trap, was introduced in 2000 ^7^, and in a relatively short time has been established as a major tool in most “omics” fields,^8–10^ due to advantages such as liquid chromatography compatible scan rate, the absence of a high-field magnet (in turn eliminating the need for cryogens), and ultrahigh *R*_p_ that has been enhanced in the last years,^11,12^ turning it into a major alternative for the most demanding MS analysis. Orbitrap FT-MS has appeared in the course of attempting to employ the Fourier transform used in FT-ICR in other trapping devices; hence FT-ICR and Orbitraps share a number of similar features. In both analyzers, the ions are trapped in ultrahigh vacuum to ensure very long free path (tens or even hundreds of kilometers). Ion detection in both instruments is also based on measuring the image charge induced by coherent motion of ions, and the use of FT of the time domain signal to generate the frequency and then mass spectra. A major advance in Orbitrap design has been recently presented by Makarov and co-workers^13^ in which a compact, high-field Orbitrap cell (Fig. S1†) showed substantial increase in *R*_p_. Later, a high-performance analyzer (that we have defined as the “MegaOrbitrap”) was shown to achieve an *R*_p_ close to or even above 1 000 000 with transients as short as 3 s.^14^

In this work, we have tested the ability of a Thermo Scientific Orbitrap Elite instrument modified with a “MegaOrbitrap” analyzer to provide *R*_p_ in excess of 1 000 000 at *m/z* 200 for accurate petroleomics analysis. To ensure that indeed proper petroleomics data is provided, the MegaOrbitrap data was compared to that obtained with a 7.2 T FT-ICR MS used for many years in our laboratory and extensively tested with acceptable performance in a variety of petroleomics studies.^15–20^

## Experimental section

South America crude oil samples were provided through collaboration with the Brazilian oil company – Petrobras (Rio de Janeiro – Brazil). The reagents used were toluene (HPLC grade, J.T. Baker, Mexico City, Mexico) and methanol (HPLC grade, Merck SA, Rio de Janeiro, Brazil) for sample dilution in a 1 : 1 (v/v) mixture (for positive and negative ion detection) with a final concentration of 1 mg mL^−1^ (in triplicate). The samples were directly infused by electrospray at a flow rate of 5 μL min^−1^ into the modified Orbitrap Elite instrument (Thermo Fisher Scientific) equipped with enhanced FT (eFT).^21^ The electrospray capillary was held at 4.0 kV for positive mode (−3.5 kV for negative mode), with transfer tube held at 280 °C and S-Lens 50 V. The ion optics were tuned to provide an optimal signal for the *m*/*z* 200–1600 range, average of 100 microscans and an AGC target of 5 × 10^5^ was used in all experiments. External calibrations were performed in both ionization modes using Pierce LTQ Velos ESI Positive Ion Calibration Solution and Negative Ion Calibration Solution (Thermo Fisher Scientific). Similar conditions were used in our 7.2 T LTQ FT Ultra (Thermo Fisher Scientific), which worked with stitched transients, *i.e.*, transients of a window of *m*/*z* stitched together to form each spectrum. PetroMS software was used to process the acquired data allowing unambiguous assignment of molecular formula. The data processing was done through the following steps: (1) the assignment of *m*/*z* for each spectrum signal; (2) automatic allocation of the optimal threshold for the noise intensity of each individual spectrum; (3) internal calibration of spectrum by homologues series using the most intense class; (4) assignment of molecular formula for each signal by comparing experimental *m*/*z* with a theoretical *m*/*z* database for possible crude oil constituents and (5) solving of dubieties on molecular formula assignments by confirming the isotopic pattern and comparison with homologous series.

## Results and discussion

**Fig. 1:**
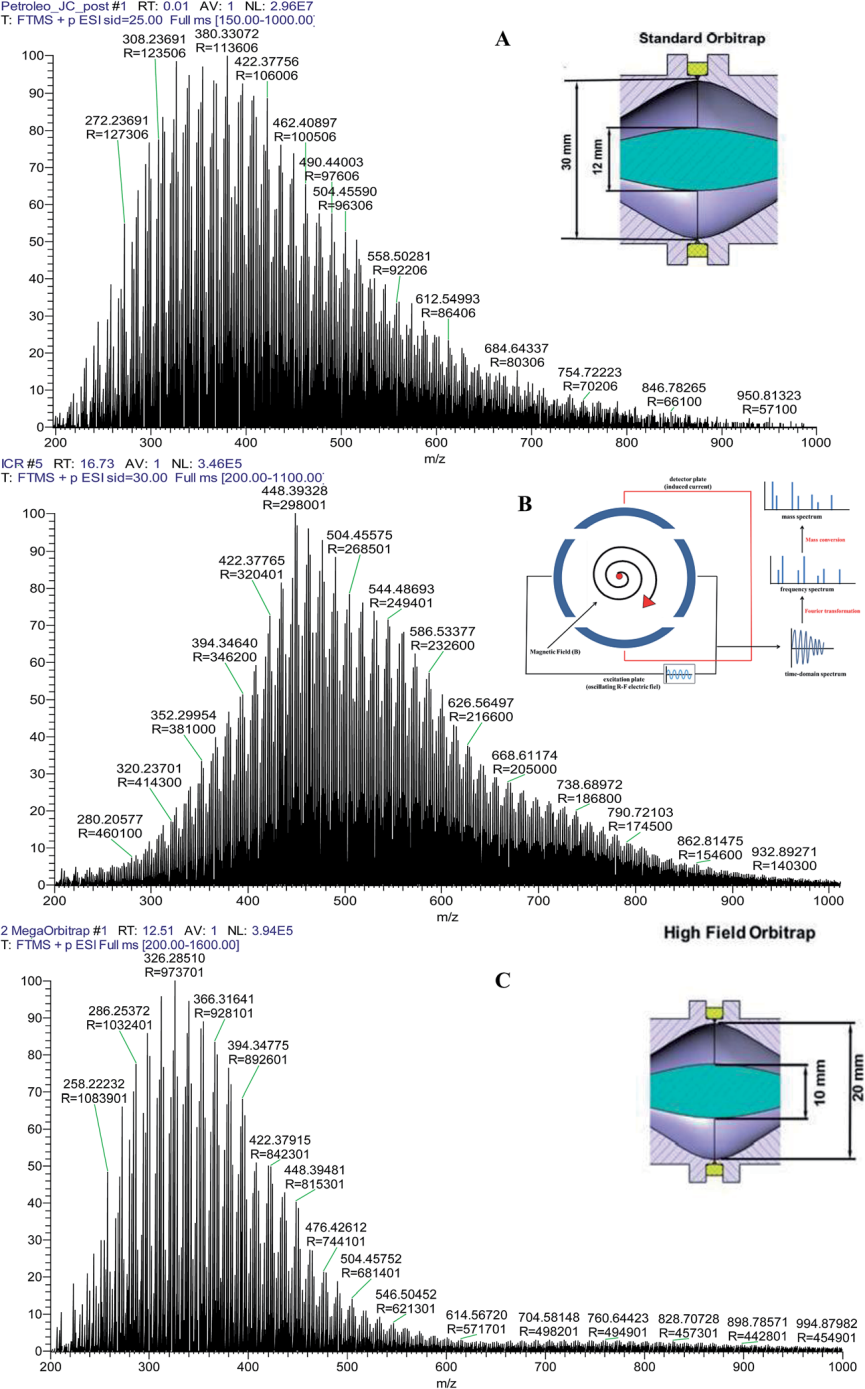
ESI(+) mass spectrum of a typical crude oil sample obtained by using the (A) standard Orbitrap, (B) 7.2 T FT-ICR and (C) the MegaOrbitrap.

Fig. 1 illustrates the ESI(+) broadband mass spectra of a typical crude oil sample obtained using each of the following analyzers: the standard Orbitrap (A), the 7.2 T FT-ICR (B) and the MegaOrbitrap (C). The selected sample was a most representative crude oil sample, one that has for many years been used in our laboratory for the calibration and performance tests for our 7.2 T FT-ICR MS instrument prior to petroleomics studies.^16^ The noise cutoff level was automatically calculated by the software and was equal to 1% of the base peak intensity. In the full *m*/*z* 200−1000 range, the total number of peaks above the noise cutoff level was 5190 for ICR and 4501 for the MegaOrbitrap.

Using a 3 s transient^14^ leads to a nominal *R*_p_ of about 340 000 at *m*/*z* 400 for the FT-ICR, and as much as 1 000 000 at *m*/*z* 200 and 840 000 at *m*/*z* 400 for the MegaOrbitrap. Note also in Fig. 1 that the Gaussian distribution of ions for the FT-ICR is shifted to higher *m*/*z* as compared to the standard Orbitrap and MegaOrbitrap analyzers. This shift likely results from FT-ICR ion optics for ion transfer from its linear trap to the ICR cell that induces a bias against low *m*/*z* ions.^26^

**Fig. 2:**
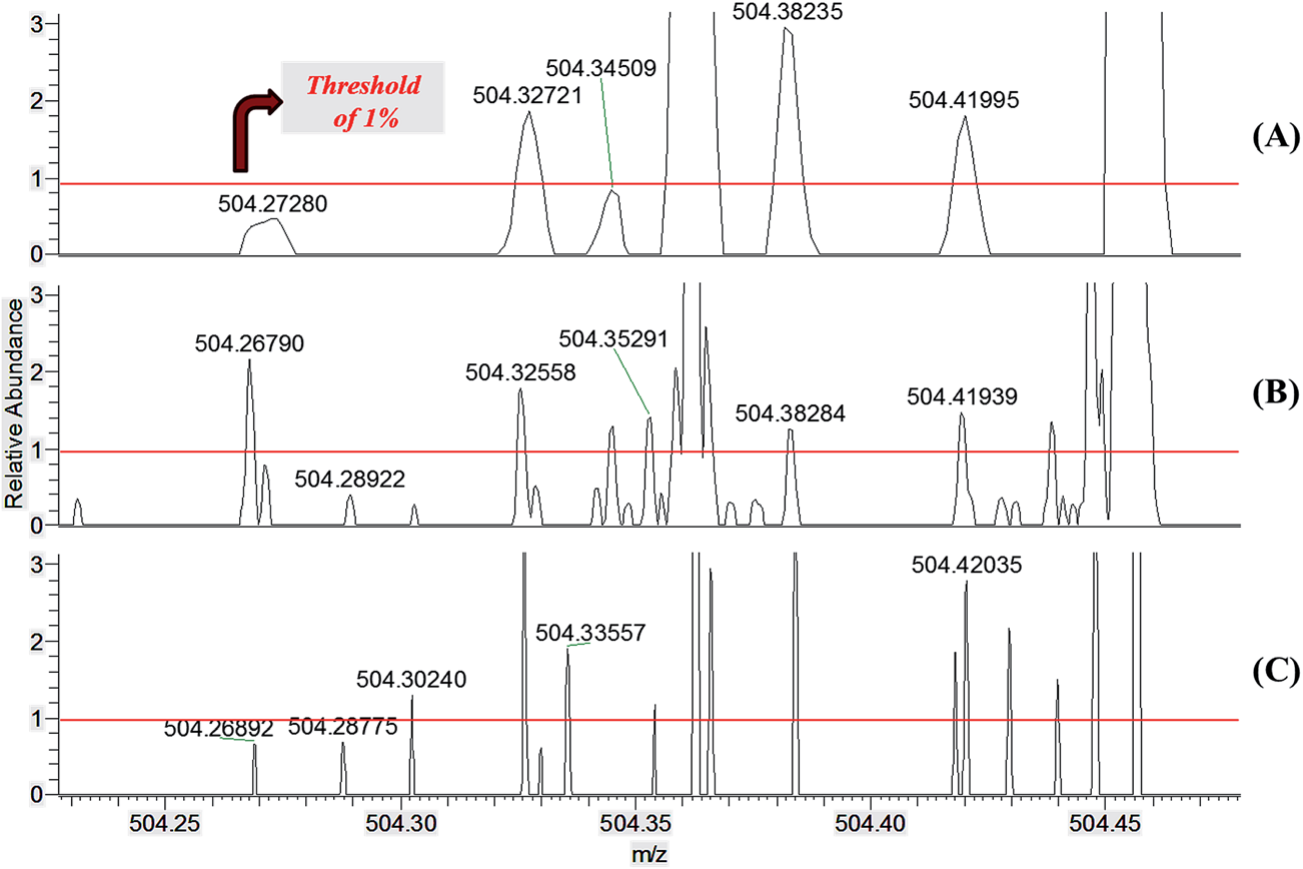
Enlargement of the ESI(+) MS data from Fig. 1 around *m*/*z* 504 for the (A) standard Orbitrap, (B) 7.2 T FT-ICR and (C) the MegaOrbitrap.

To illustrate cut off levels, noise and peak broadness and symmetry, Fig. 2 shows expanded views for the *m*/*z* 504 region of mass spectra obtained using the three instruments whereas Table S1† provides major ion assignments.

**Table 1:** Figures of merit for the three analyzers as measured from their ESI(+)-MS data

Parameter	Standard Orbitrap	7.2 T FT-ICR	MegaOrbitrap
Signal-to-noise	1905	637	1933
Dynamic range	141 : 1	125 : 1	127 : 1
Spectral error^a^	13 ± 10	15 ± 10	15 ± 11
Transient duration	0.51 s	3 s	3 s
Mass accuracy^b^	1.01 ± 0.23 ppm	0.81 ± 0.10 ppm	0.93 ± 0.15 ppm
*R*_p_	109 902 ± 752, *m*/*z* 400	337 100 ± 439, *m*/*z* 400	841 004 ± 339, *m*/*z* 400
No. of identified classes (+ESI)	1	4	4

a M + 1.

b Monoisotopic masses.

Fig. 2 provides an overall view of the spectra quality data. In the illustrative *m*/*z* 504.26–504.46 range, the total number of ions above 1% of relative intensity is 5 for the standard Orbitrap, 13 for the 7.2 T FT-ICR, and 13 for the MegaOrbitrap. Note also that for the MegaOrbitrap the signals are much more resolved.

**Fig. 3:**
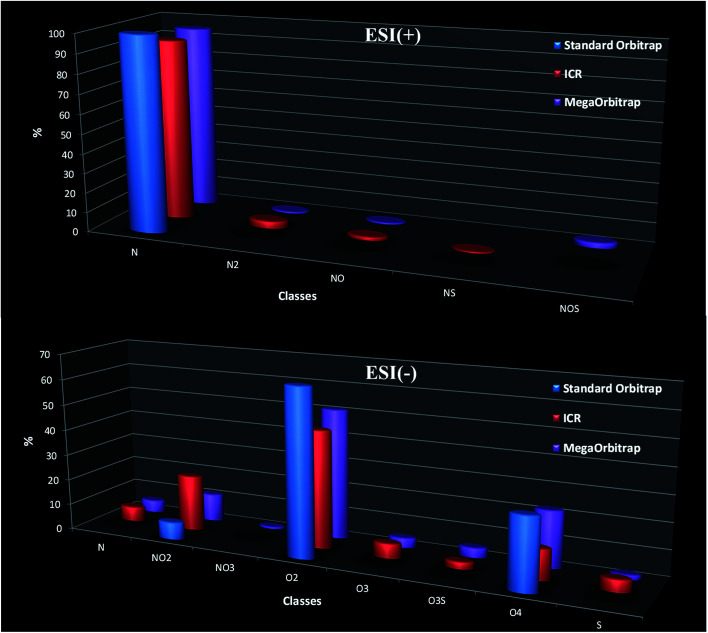
Class distributions from ESI(+) and ESI(−) MS data as determined from data collected with the standard Orbitrap, the 7.2 T FT-ICR and the MegaOrbitrap.

**Fig. 4:**
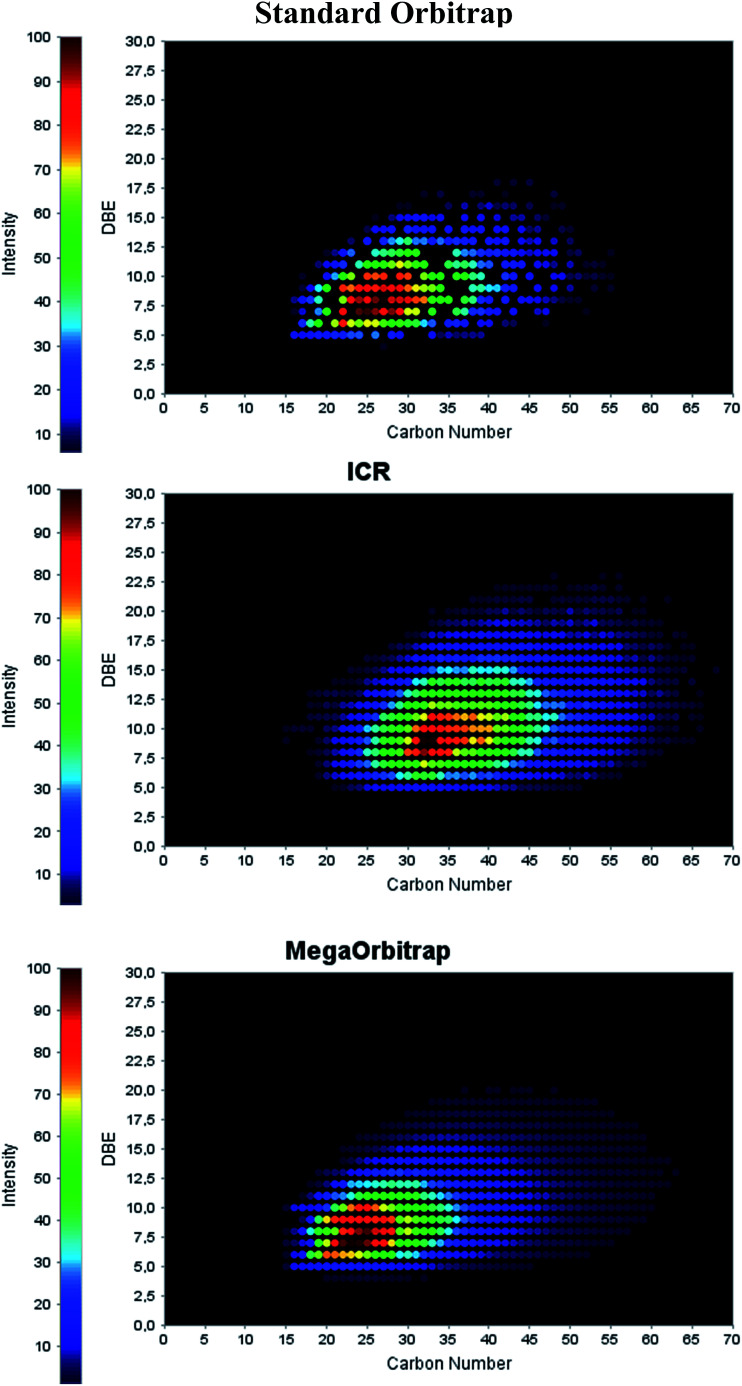
Carbon number (C_*n*_) *versus* DBE for the N class as determined from ESI(+) MS data collected with the standard Orbitrap, the 7.2 T FT-ICR and the MegaOrbitrap.

In petroleomics MS, graphic tools such as class distributions, van Krevelen and Kendrick diagrams have been extensively used for a better visualization and geochemical interpretation of the data.^16^Fig. 3 compares the performance of the three analyzers in the assignment of the N class distributions in the crude oil sample, whereas Fig. 4 compares the carbon number (C_*n*_) *versus* double bound equivalent (DBE) distribution specifically for the N class.

Note in Fig. 3 the poor performance of the standard Orbitrap (512 ms transient → 140k at *m*/*z* 200) since it was only able to attribute the major N class while missing the other less abundant classes. But the MegaOrbitrap displayed similar class attribution as that of the 7.2 T FT-ICR. In fact, it seems that the 7.2 T FT-ICR has attributed one of the minor class as NS whereas the MegaOrbitrap attributed it as NOS.

Note now in Fig. 4 that again the performance of the standard Orbitrap was poor whereas that of both the 7.2 T FT-ICR and MegaOrbitrap seem to be excellent and similar. For the standard Orbitrap a quite irregular profile was detected with many missing dots and gaps, due to insufficient *R*_p_ and/or accuracy. But both the 7.2 T FT-ICR and MegaOrbitrap show quite regular and complete C_*n*_ × DBE distributions. Note also that, as already discussed and shown in Fig. 1, the center of mass for the 7.2 T FT-ICR (around C35) is artificially shifted to higher *m*/*z* as compared to that of both the standard Orbitrap and MegaOrbitrap (C25).

Fig. 3 compares the analyzers in their ability to correctly attribute classes of ions in the ESI(−) ion mode. Note again, as for ESI(+), the poor performance of the standard Orbitrap since it was only able to attribute three major classes (NO_2_, O_2_ and O_4_) while missing several other less abundant classes and with probably misassignments of relative abundances due to class overlaps. But both the 7.2 T FT-ICR and the MegaOrbitrap display nearly the same class attributions. That is, in fact eight classes were attributed by the MegaOrbitrap whereas the 7.2 T FT-ICR seems to have missed the NO_3_ class.

An appropriate way to evaluate analyzers consists in comparing their figures of merit.^22^ Here, as Fig. S2† illustrates, a set of seven figures of merit have been compared. Previous comparisons of FT-ICR and Orbitraps of such figures of merit in proteomic studies have been done.^13,23–26^

### Signal to noise

**Fig. 5:**
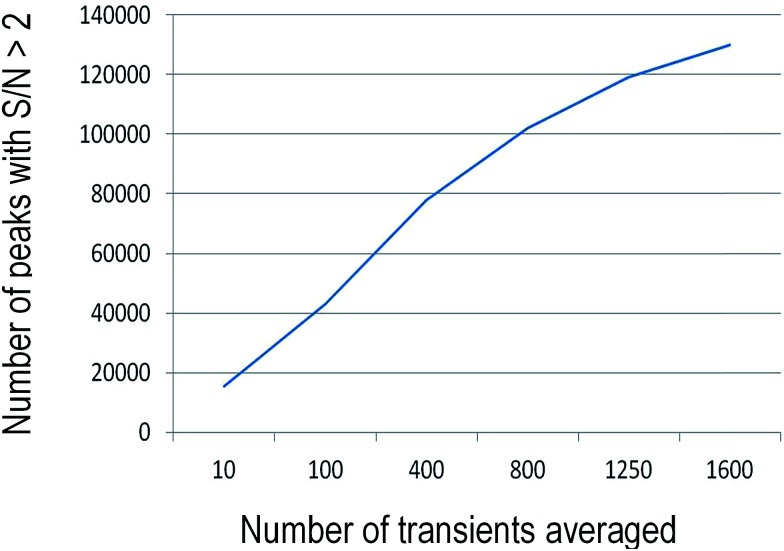
Number of detected ions with S/N > 2 according to number of transients averaged of MegaOrbitrap.

In MS, S/N ratios are known to be directly influenced by the abundance of the ions (peak intensity), while noise stay relatively constant along the used *m*/*z* window.^18^ Moreover, both signal and noise increase with acquisition time, while signal increases proportional to time, noise increases proportional to square root of it, increasing the S/N ratio with increasing acquisition time. This S/N increase can be observed by the rise in the number of peaks with S/N > 2 with averaged transients in the MegaOrbitrap data (Fig. 5).^18^ In order to compare all of analyzers, the most abundant ions of each spectra was used to obtain the S/N ratio. Fig. S3† shows the noise (N) for each signal (provided by the Thermo Scientific Xcalibur software) and the S/N measurements for the three analyzers. Note that both the standard and MegaOrbitrap are found to display quite similar performance (S/N = 1905 and 1933 respectively), which was much superior to that of the 7.2 FT-ICR (S/N = 637).

### Dynamic range

Dynamic range (DR) is normally defined as the ratio between the largest and smallest ion peaks in the spectrum that can have their *m*/*z* values accurately measured and assigned.^18^ DR was calculated in the range of *m*/*z* 100–1500 (Fig. S4†). First, it was needed to find the minimum relative peak intensity with reliable molecular formula assignment. Assuming that the chemical noise has a normal distribution with mean (*μ*) equal zero, it was therefore possible to calculate the population standard deviation obtaining the value of sigma (*σ*) which can be used to determine the threshold of result's reliability. For this threshold, we use the value of 3*σ* providing a reliability of 99.75%. For the standard Orbitrap, note that the lowest attributed ion has a relative peak intensity equal to 0.701% when compared to the highest peak in the range, which gives a DR of 141 : 1. For the 7.2 T FT-ICR, the lowest peak has a relative intensity of 0.796% in comparison to the most intense peak, which gives a DR of 125 : 1. For the MegaOrbitrap, a relative intensity of 0.784% is obtained, which leads to a DR of 127 : 1. These results show that both the standard and the MegaOrbitrap provide DR similar to the 7.2 FT-ICR for petroleomics analysis.

**Fig. 6:**
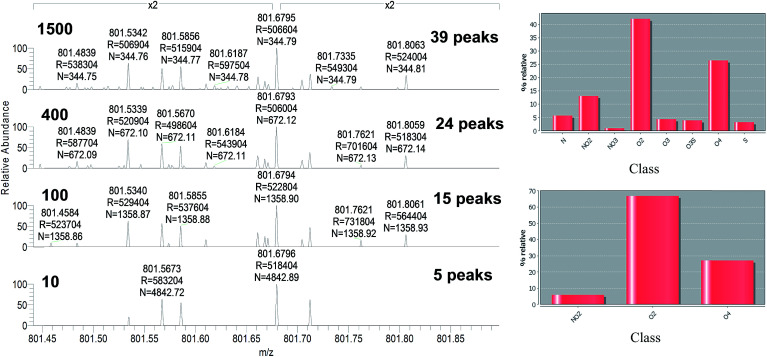
Higher dynamic range (more classes identified) by averaging more transients with MegaOrbitrap in negative mode.

Additionally, the DR also increases with the number of averaged transients, since the S/N ratio increases with acquisition time, and more ions corresponding to more classes can be assigned (Fig. 6).

### Spectral error (SE)

Spectral error in MS is known to vary according to the relative peak intensity as well as *m*/*z*. The spectral error is defined as:
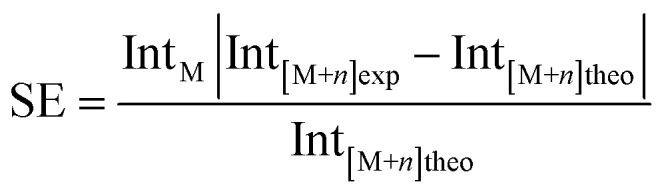
where Int_M_ is the intensity of the monoisotopic ion, |Int_[M+*n*]exp_ − Int_[M+*n*]theo_| is the absolute value of the difference between the experimental and theoretical intensities of the isotopologue ion. This theoretical value is calculated based on the attributed formula, with ^13^C natural abundance being 1109% relative to ^12^C. Fig. S5† shows the expansion of the mass spectra in the *m*/*z* 560.4–561.6 range, and the assignment of the C_40_H_66_N and its isotopologue C_39_^13^CH_66_N ions. Table S2† shows the average SE for all attributed classes that showed detectable isotopologue ^13^C ions. Similar spectral errors were observed for all three instruments, in both positive and negative modes: 13% for standard Orbitrap, 15% for both, MegaOrbitrap and 7.2 T FT-ICR.

### Scan speed

The scan speed was measured based on the transient length of each instrument, and are compared in Fig. S6.† The standard Orbitrap operating at its maximum transient length of 0.51 s provided an *R*_p_ of 140 000 at *m*/*z* 200. When comparing their abilities to provide an *R*_p_ of 400 000 at *m/z* 400, we observe that the MegaOrbitrap achieves such *R*_p_ at half of the transient time (1.5 s) needed for the 7.2 T FT-ICR (3 s).

### Mass accuracy

Fig. S7† shows a collection of ions in the *m*/*z* range of 510–520 and their respective mass accuracies measured using the three analyzers. For example, note to the *m*/*z* 516 that the mass errors substantially decrease from the standard Orbitrap (1.1 ppm) to the 7.2 T FT-ICR (0.60 ppm) and then to the MegaOrbitrap (0.44 ppm). For petroleomics investigations,^17,27^ an accuracy of 1 ppm or ideally lower than 1 ppm has been shown to be essential and, therefore, both the 7.2 T FT-ICR but more effectively the MegaOrbitrap with an accuracy of *ca.* 450 ppb have been able to fulfill this important requirement. The cooling system (chiller) to Orbitrap Elite is more efficient.

### Resolving power

**Fig. 7:**
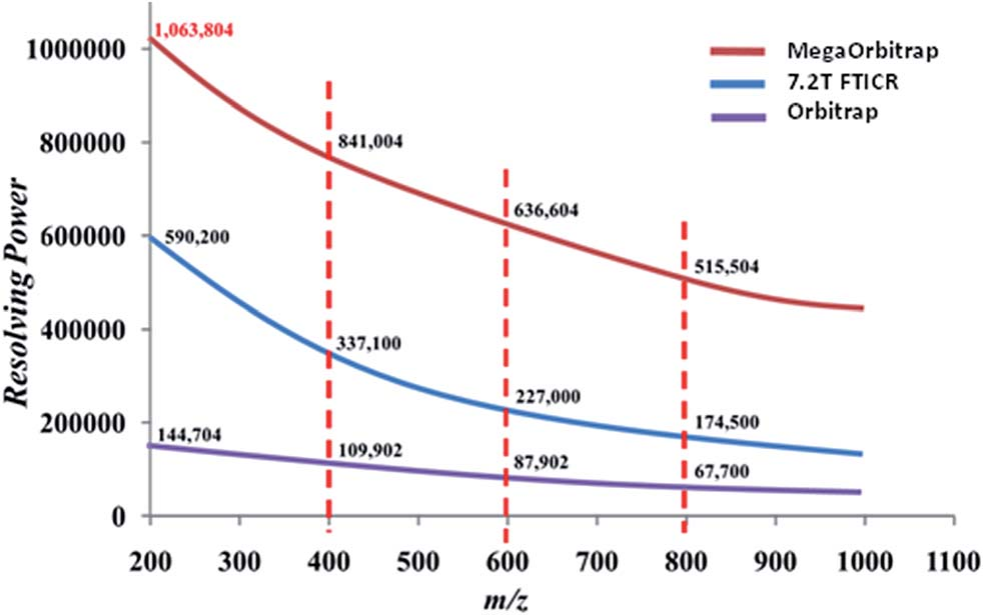
*R*_p_ obtained from the ESI(+)-MS data for the analysis of a typical crude oil sample in the 7.2 T FT-ICR, standard Orbitrap and the MegaOrbitrap.

As Fig. S7† also shows the *R*_p_ measured for the theoretical *m*/*z* 516.45638 increases considerably from the standard Orbitrap (90 837) to the 7.2 T FT-ICR (265 153), and then to the MegaOrbitrap (665 404). However, as sufficient *R*_p_ to resolve isobaric doublets is needed most often in the *m*/*z* 200–1000 range for petroleomics studies, the *R*_p_ of the three analyzers were also compared along this range (Fig. 7). Table S3† shows also the minimum *R*_p_ as a function of mass needed to resolve the isobaric C_3_ and SH_4_ doublet which is separation by as little as 0.0034 Da.

When comparing the results summarized in Fig. 7 and Table S3,† we see for instance that at *m*/*z* 200 all analyzers are able to resolve C_3_/SH_4_ doublets. But at *m*/*z* 400, only the 7.2 T FT-ICR and the MegaOrbitrap perform well, whereas at *m*/*z* 600 or higher, only the MegaOrbitrap is able to properly resolve this crucial C_3_/SH_4_ isobaric doublet commonly dealt with in petroleomics MS.

Table 1 summarizes the comparison for all six figures of merit between the three analyzers, permitting an overall comparison. In general, the standard Orbitrap shows poor performance for petroleomics investigations although for less complex samples such as crude oil distillates, reasonable performance for standard Orbitraps in petroleomics studies has been reported.^12,28–30^ But for the reference crude oil sample selected for this study, with a typical and quite complex composition, both the MegaOrbitrap and the 7.2 T FT-ICR performs quite well in terms of the seven figures of merit investigated herein.

## Conclusions

The continuous pursuit for higher *R*_p_ and accuracy in mass spectrometry has led to superb performance of FT-ICR but at the cost of increasing magnetic fields, sizes and maintenance demands, and cost. Although FT-ICR MS systems that deliver superior performance are currently available, using a 7.2 T FT-ICR MS system that has been for many years used and probed to provide reliable petroleomics data in a variety of applications and samples, this study has shown that sufficiently accurate, precise, and fast petroleomics analysis can be performed in the less demanding MegaOrbitrap mass analyzers with *R*_p_ exceeding 1 000 000 at *m/z* 200 and accuracy in order of ppb. Another beneficial feature of the MegaOrbitrap for petroleomics studies that is currently been tested is also to take advantage of the superior *R*_p_ to confirm class attributions *via* fine isotope signatures for A + 1 and A + 2 peaks. After decades of only having a single option for direct infusion ultra-high resolution analysis, the MegaOrbitrap now offers an attractive and effective alternative for petroleomics studies.

## Funding sources

Centro de Pesquisas da Petrobras (CENPES).

## Conflicts of interest

The authors declare the following competing financial interest(s): three of the authors (Eduard Denisov, Eugen Damoc and Alexander Makarov) are employees of Thermo Fisher Scientific, which manufactures and sells Orbitrap-based mass spectrometers.

## Supplementary Material

RA-008-C7RA12509G-s001
