# Supplementary material for: Petroleomics via Orbitrap mass spectrometry with resolving power above 1 000 000 at m/z 200
Source: RSC Adv. 2018 Feb 7;8(11):6183–91. doi: 10.1039/c7ra12509g (PMC9078259; doi:10.1039/c7ra12509g)
Supplement: RA-008-C7RA12509G-s001 [file RA-008-C7RA12509G-s001.pdf]

Electronic Supplementary Material (ESI) for RSC Advances.
This journal is © The Royal Society of Chemistry 2018

# Supporting Information

**Title:** Petroleomics via Orbitrap mass spectrometry with resolving power above 1,000,000

**Authors:** *Eduardo M. Schmidt[†, ‡], Marcos A. Pudenzi[†], Jandyson M. Santos[†], Celio F. F. Angolini[†], Rosana C. L. Pereira[£], Ygor Rocha[£], Eduard Denisov[¥], Eugen Damoc[¥], Alexander Makarov[¥*], Marcos N. Eberlin[*,†]*

[†] ThoMSon Mass Spectrometry Laboratory, Institute of Chemistry, University of Campinas, UNICAMP, 13083-725, Campinas, SP, Brazil

[‡] Nova Analítica Importações e Exportações LTDA, 04131-000, São Paulo, SP, Brazil.

[£] Petróleo Brasileiro S/A, PETROBRAS, CENPES, 21941-915, Rio de Janeiro, RJ, Brazil

[¥] Thermo Fisher Scientific, Hanna-Kunath-Strasse 11, 28199, Bremen, Germany.

**Corresponding Authors:** *eberlin@iqm.unicamp.br (M.N.E); alexander.makarov@thermofisher.com (A.M).

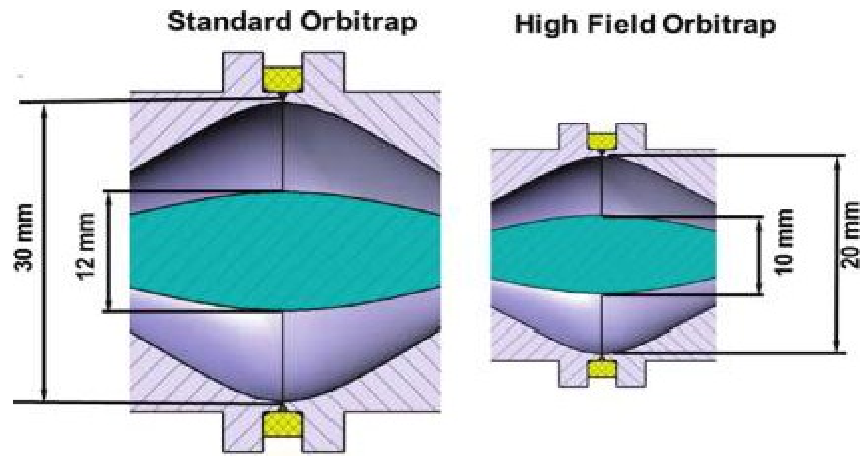


**Figure S1:** The standard Orbitrap geometry (left) and that of a compact high-field Orbitrap (right) analyzer. The outer electrodes are separated by a quartz ring and used for detection and therefore are maintained at the virtual ground of the preamplifier, whereas the central electrode is used for trapping at a voltage – Ur (Ur > 0 for positive ions). Courtesy of © Thermo Fisher Scientific (Bremen) GmbH.

**Table S1:** Molecular formula assigned to ions detected and separated by the standard Orbitrap, the 7.2 T FT-ICR and the MegaOrbitrap analyzers in the *m/z* 504.26 – 504.46 range.

| Molecular Formula | Theorical Mass | Mass Analyzer | Experimental Mass | Absolute intensity | Error (ppm) | Resolving Power |
|---|---|---|---|---|---|---|
| **$C_{38}H_{34}N$** | 504.26858 | Std. Orbitrap | X | X | X | X |
| | | FT-ICR | 504.26790 | 2346.4 | -1.342 | 252,100 |
| | | MegaOrbitrap | X | X | X | X |
| **$C_{36}H_{42}NO$** | 504.32609 | Std. Orbitrap | 504.32721 | 4294 | 2.218 | 73,900 |
| | | FT-ICR | 504.32558 | 4342.7 | -1.014 | 234,700 |
| | | MegaOrbitrap | 504.32635 | 15873.7 | 0.513 | 665,004 |
| **$C_{33}H_{46}NOS$** | 504.32946 | Std. Orbitrap | X | X | X | X |
| | | FT-ICR | X | X | X | X |
| | | MegaOrbitrap | 504.32986 | 2961.1 | 0.797 | 630,456 |
| **$C_{27}H_{54}NOS_3$** | 504.33620 | Std. Orbitrap | X | X | X | X |
| | | FT-ICR | X | X | X | X |
| | | MegaOrbitrap | 504.33557 | 9152.9 | -1.257 | 621,404 |
| **$C_{31}H_{52}OS_2$** | 504.34541 | Std. Orbitrap | 504.34509 | 2205.9 | -0.633 | 75,000 |
| | | FT-ICR | 504.34489 | 2260.7 | -1.029 | 248,400 |
| | | MegaOrbitrap | X | X | X | X |
| **$C_{37}H_{46}N$** | 504.36248 | Std. Orbitrap | 504.36185 | 33785 | -1.243 | 91,502 |
| | | FT-ICR | 504.36186 | 61057.8 | -1.223 | 266,600 |
| | | MegaOrbitrap | 504.36279 | 106921.5 | 0.621 | 634,604 |
| **$C_{34}H_{50}NS$** | 504.36585 | Std. Orbitrap | X | X | X | X |
| | | FT-ICR | 504.36503 | 5488.8 | -1.621 | 273,600 |
| | | MegaOrbitrap | 504.36603 | 14255.1 | 0.361 | 664,404 |

| | | | | | | |
|---|---|---|---|---|---|---|
| **$C_{34}H_{50}NO_2$** | 504.38361 | Std. Orbitrap | 504.38235 | 5622.7 | -2.491 | 86,602 |
| | | FT-ICR | 504.38284 | 2683 | -1.519 | 220,900 |
| | | MegaOrbitrap | 504.38394 | 21729.2 | 0.662 | 668,904 |
| **$C_{35}H_{54}NO$** | 504.41999 | Std. Orbitrap | 504.41995 | 4192.1 | -0.083 | 77,400 |
| | | FT-ICR | 504.41939 | 3695.9 | -1.193 | 229,100 |
| | | MegaOrbitrap | 504.42035 | 13535.1 | 0.710 | 692,604 |
| **$C_{31}H_{56}N_2O_3$** | 504.42855 | Std. Orbitrap | X | X | X | X |
| | | FT-ICR | X | X | X | X |
| | | MegaOrbitrap | 504.42947 | 10397.3 | 1.834 | 654,904 |
| **$C_{30}H_{64}OS_2$** | 504.43931 | Std. Orbitrap | X | X | X | X |
| | | FT-ICR | 504.43856 | 1063.5 | -1.486 | 284,300 |
| | | MegaOrbitrap | 504.43976 | 7245.1 | 0.893 | 670,604 |
| **$C_{32}H_{60}N_2S$** | 504.44717 | Std. Orbitrap | X | X | X | X |
| | | FT-ICR | 504.44688 | 12948.9 | -0.579 | 251,500 |
| | | MegaOrbitrap | 504.44775 | 28837.3 | 1.146 | 665,604 |
| **$C_{36}H_{58}N$** | 504.45637 | Std. Orbitrap | 504.45572 | 172385.9 | -1.303 | 96,506 |
| | | FT-ICR | 504.45573 | 199385.4 | -1.283 | 268,401 |
| | | MegaOrbitrap | 504.45670 | 479846.7 | 0.640 | 695,501 |

X= Not detected in this condition

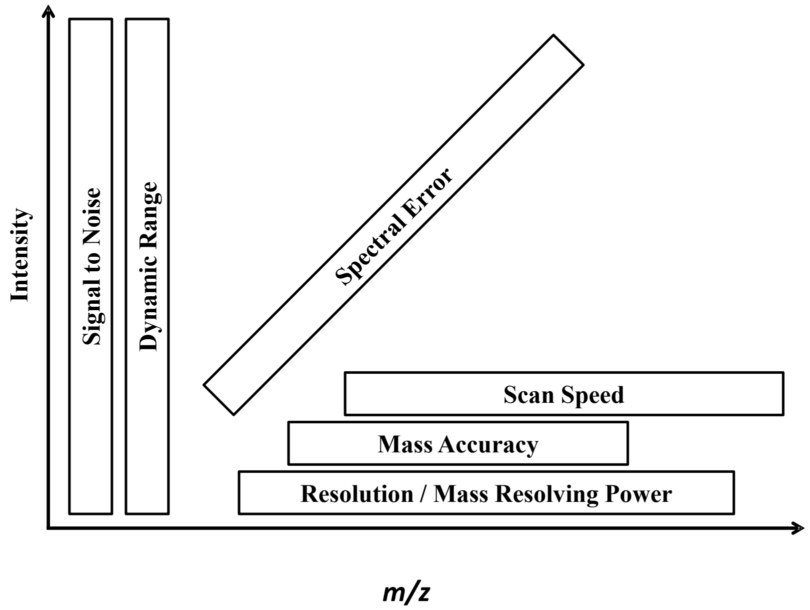


**Figure S2:** Major figures of merit used in mass spectrometry and their correlation with *m/z* values and peak intensity.

**Signal to Noise ***

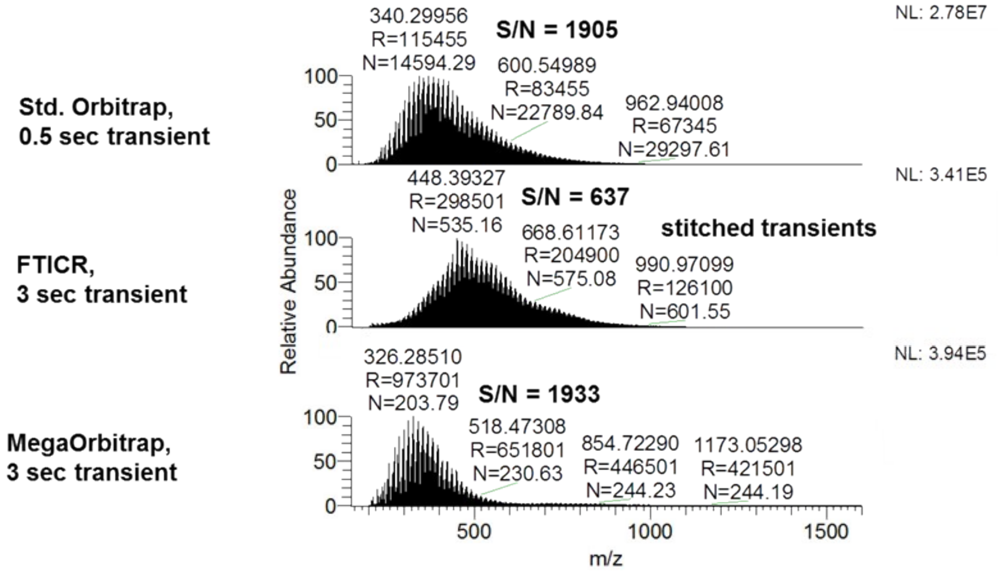


**Figure S3*:** Signal-to-Noise ratio obtained from the analysis of a typical crude oil sample in Standard Orbitrap, FT-ICR, and MegaOrbitrap.

**Dynamic Range**

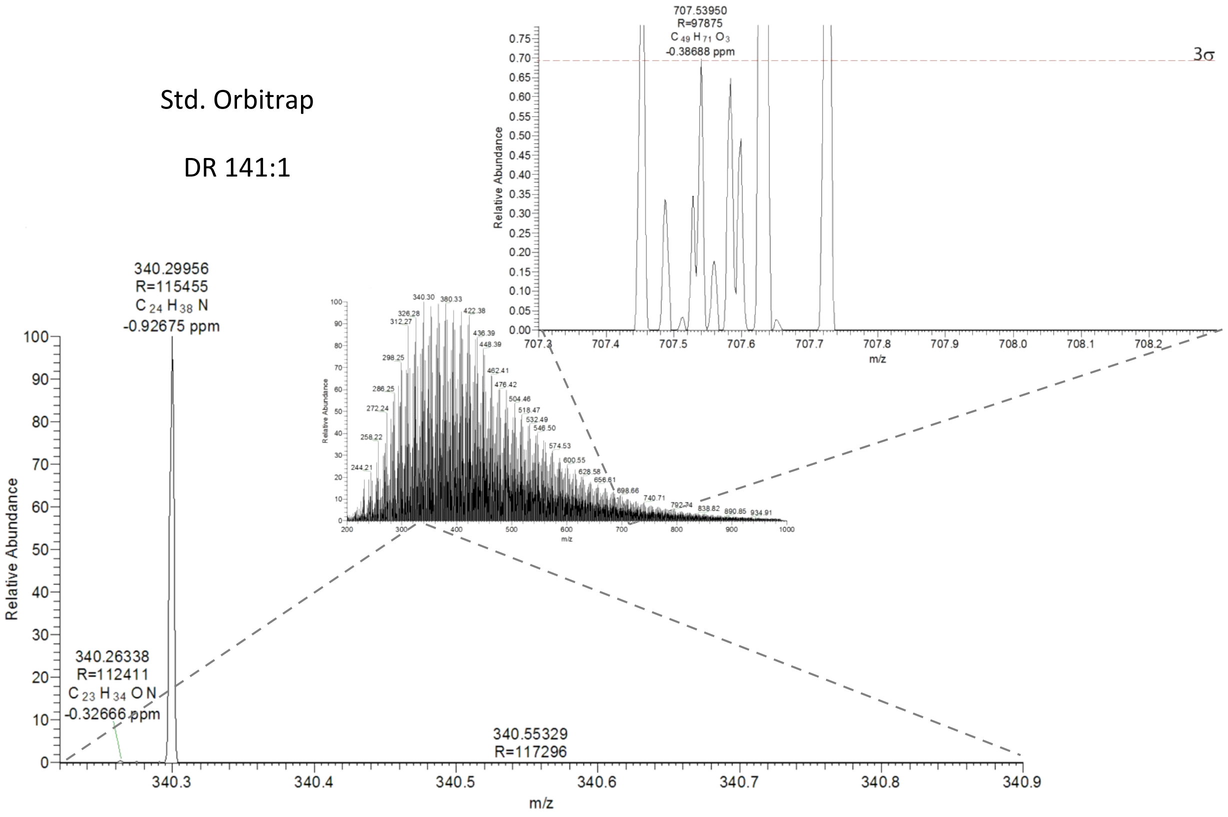
Std. Orbitrap
DR 141:1
340.29956
R=115455
C 24 H 38 N
-0.92675 ppm
340.26338
R=112411
C 23 H 34 O N
-0.32666 ppm
340.55329
R=117296
Relative Abundance
m/z
707.53950
R=97875
C 49 H 71 O 3
-0.38688 ppm
3σ

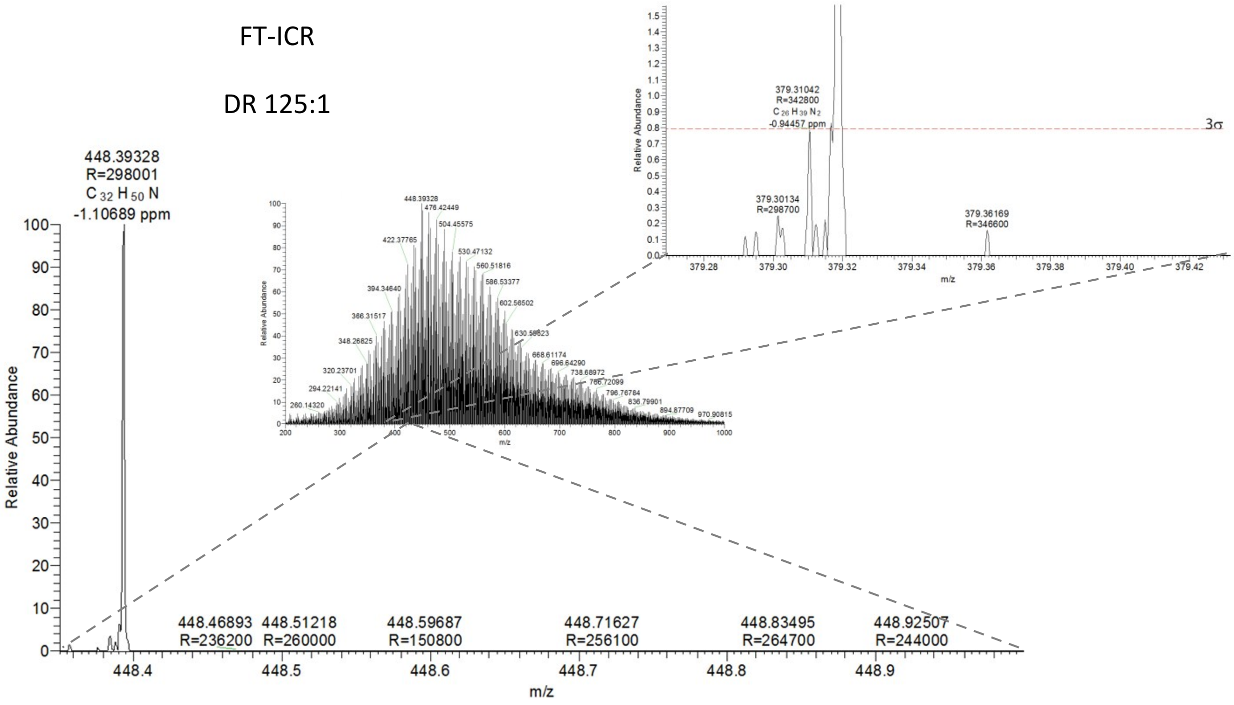
FT-ICR
DR 125:1
448.39328
R=298001
C 32 H 50 N
-1.10689 ppm
448.46893
R=236200
448.51218
R=260000
448.59687
R=150800
448.71627
R=256100
448.83495
R=264700
448.92507
R=244000
Relative Abundance
m/z
379.31042
R=342800
C 26 H 39 N 2
-0.94457 ppm
379.30134
R=298700
379.36169
R=346600
3σ

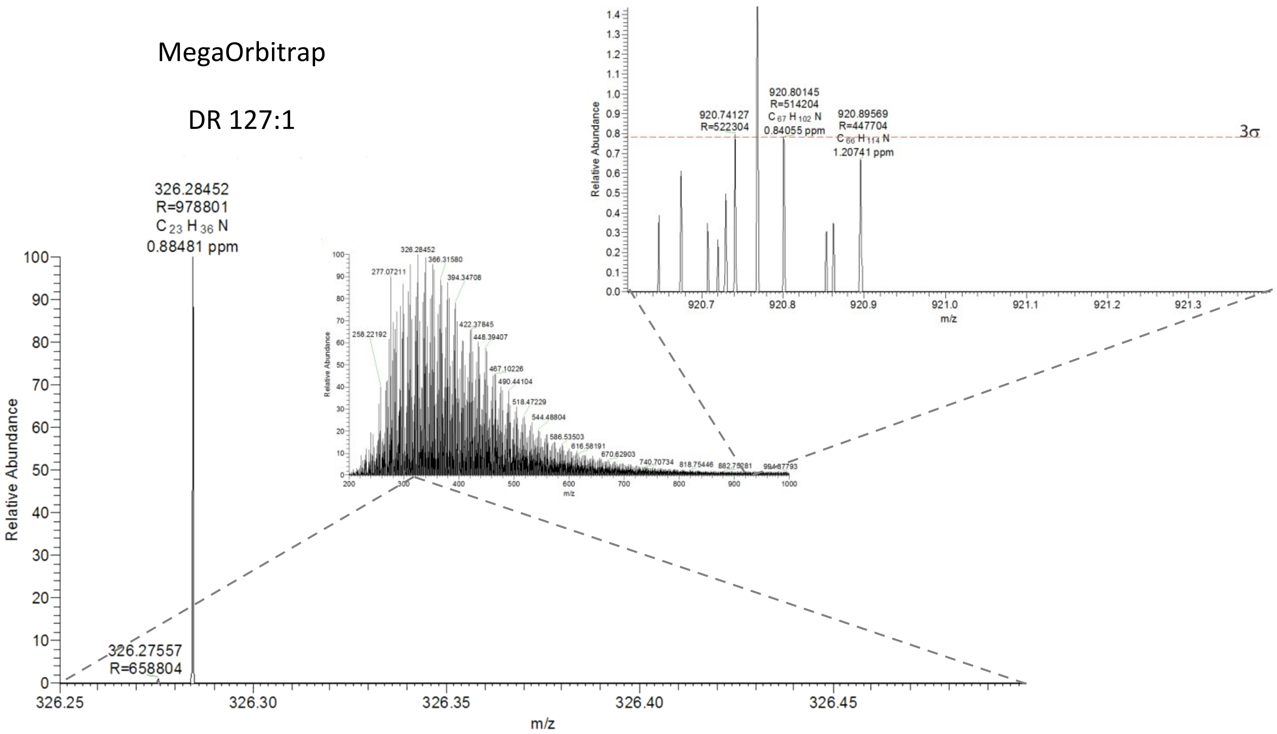


**Figure S4*:** Calculated Dynamic Range in the analysis of a typical crude oil sample in Standard Orbitrap, FT-ICR and MegaOrbitrap.

**Spectral Error**

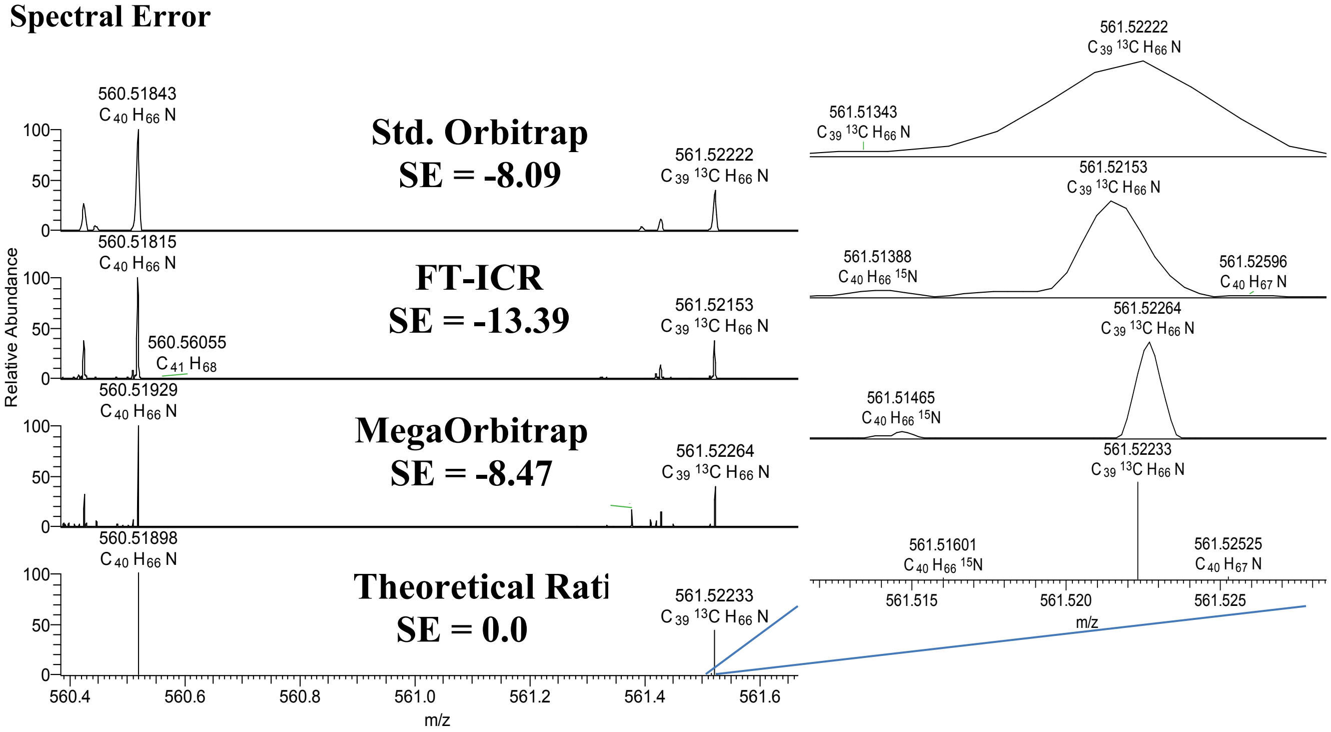


**Figure S5*:** Spectral Error (SE) for $C_{40}H_{66}N$ and its isotope $C_{39}{}^{13}CH_{66}N$ obtained from the analysis of a typical crude oil sample by Standard Orbitrap, FT-ICR, and MegaOrbitrap.

**Table S2*:** Mean spectral error (SE) for all molecular formula assigned obtained from the analysis of a typical crude oil sample by using the Standard Orbitrap, FT-ICR, and MegaOrbitrap. The total number of molecular formulas and attributed isotopologues is also shown.

| ***Mass Analyzer*** | **Mean spectral error (SE) (%)** | | | Molecular formula (attributed isotopologues) |
|---|---|---|---|---|
| | **M+1** | **M+2** | **Total** | |
| Standard Orbitrap | 12 ± 10 | 29 ± 8 | 13 ± 10 | 537 (512) |
| FT-ICR | 14 ± 10 | 18 ± 10 | 15 ± 10 | 2376 (1661) |
| MegaOrbitrap | 14 ± 11 | 16 ± 11 | 15 ± 11 | 1754 (1320) |

**Scan Speed**

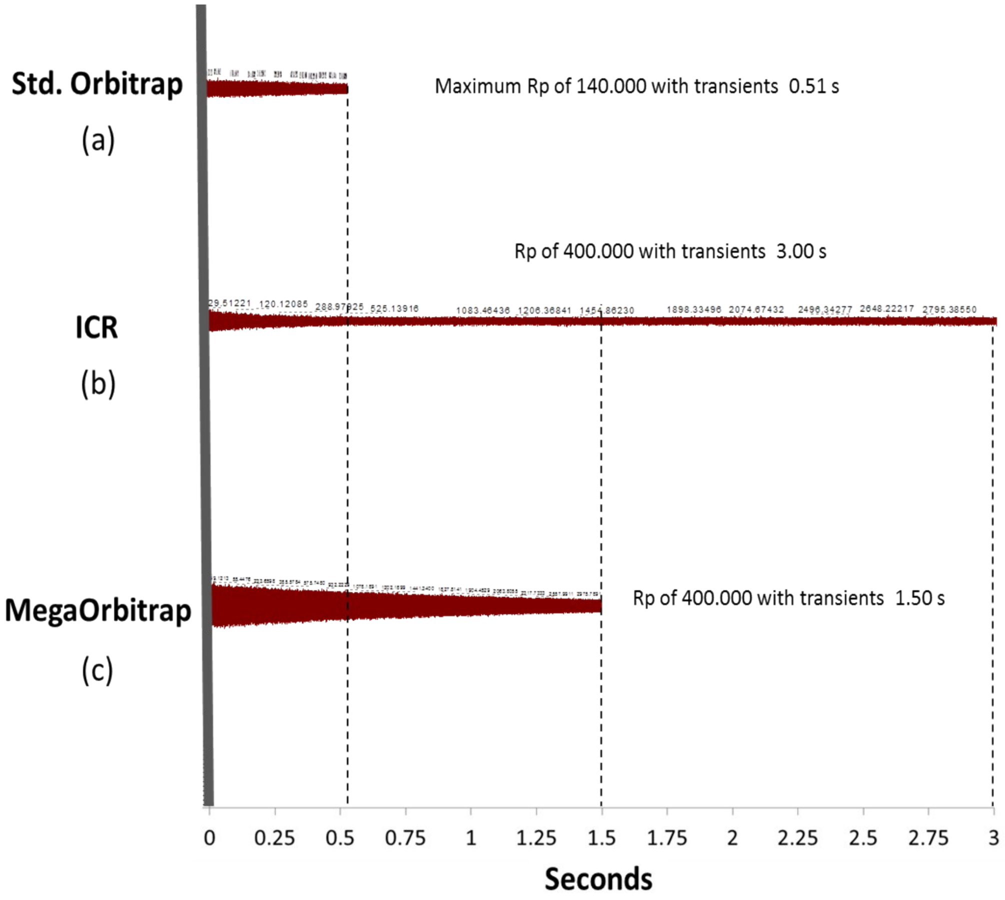


**Figure S6*:** Transients obtained for each equipment organized in the same time scale in seconds. They refer to (a) Standard Orbitrap (b) FT-ICR and (c) MegaOrbitrap

## Mass Accuracy

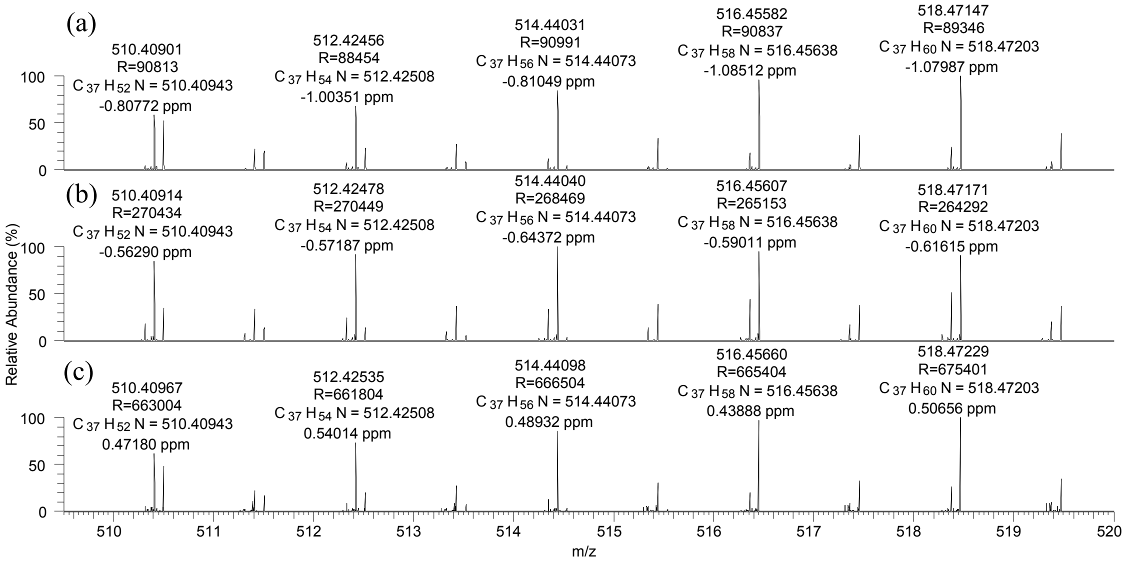


**Figure S7*:** Spectra in the same mass range showing the differences in mass accuracy and resolution between the equipments studied: (a) Standard Orbitrap (b) FT-ICR and (c) MegaOrbitrap

**Table S3*:** Minimum resolving power required for resolve compounds differing by $C_3$ and $SH_4$ at different *m/z*

| *m/z* | Minimum Resolving Power |
|---|---|
| 200 | 117647 |
| 400 | 235294 |
| 600 | 352941 |
| 800 | 470588 |
